# Carnosol inhibits inflammasome activation by directly targeting HSP90 to treat inflammasome-mediated diseases

**DOI:** 10.1038/s41419-020-2460-x

**Published:** 2020-04-20

**Authors:** Wei Shi, Guang Xu, Xiaoyan Zhan, Yuan Gao, Zhilei Wang, Shubin Fu, Nan Qin, Xiaorong Hou, Yongqiang Ai, Chunyu Wang, Tingting He, Hongbin Liu, Yuanyuan Chen, Yan Liu, Jiabo Wang, Ming Niu, Yuming Guo, Xiaohe Xiao, Zhaofang Bai

**Affiliations:** 1 China Military Institute of Chinese Materia, The Fifth Medical Centre, Chinese PLA General Hospital, Beijing, 100039 China; 2 School of Pharmacy, Jiangxi University of Traditional Chinese Medicine, Nanchang, 330004 China; 3 Integrative Medical Centre, The Fifth Medical Centre, Chinese PLA General Hospital, Beijing, 100039 China; 4 School of Traditional Chinese Medicine, Capital Medical University, Beijing, 100069 China; 5 School of Pharmacy, Chengdu University of Traditional Chinese Medicine, Chengdu, 611137 China

**Keywords:** Inflammasome, Inflammatory diseases

## Abstract

Aberrant activation of inflammasomes, a group of protein complexes, is pathogenic in a variety of metabolic and inflammation-related diseases. Here, we report that carnosol inhibits NLRP3 inflammasome activation by directly targeting heat-shock protein 90 (HSP90), which is essential for NLRP3 inflammasome activity, thereby treating inflammasome-mediated diseases. Our data demonstrate that carnosol inhibits NLRP3 inflammasome activation in primary mouse bone marrow-derived macrophages (BMDMs), THP-1 cells and human peripheral blood mononuclear cells (hPBMCs). Mechanistically, carnosol inhibits inflammasome activation by binding to HSP90 and then inhibiting its ATPase activity. In vivo, our results show that carnosol has remarkable therapeutic effects in mouse models of NLRP3 inflammasome-mediated diseases, including endotoxemia and nonalcoholic steatohepatitis (NASH). Our data also suggest that intraperitoneal administration of carnosol (120 mg/kg) once daily for two weeks is well tolerated in mice. Thus, our study reveals the inhibitory effect of carnosol on inflammasome activation and demonstrates that carnosol is a safe and effective candidate for the treatment of inflammasome-mediated diseases.

## Introduction

Inflammasomes are multiprotein complexes that can be activated by pathogen-associated molecular patterns (PAMPs) and danger-associated molecular patterns (DAMPs) to trigger the catalytic activation of caspase-1, subsequently leading to pyroptosis and the production of interleukin 1β (IL-1β) and IL-18^1–3^. Previous studies have confirmed that inflammasomes are involved in the initiation of various metabolic and inflammation-related diseases^4,5^. Pharmacological inhibitors of inflammasomes have produced remarkable therapeutic effects in animal models of various human diseases^6–11^. Thus, inflammasomes are widely considered to be new targets for the treatment of many diseases.

NOD-like receptor (NLR) family members including NLRP1, NLRP3, and NLRC4, as well as the cytosolic receptor AIM2, have been shown to form inflammasomes^12–16^. Among them, the NLRP3 inflammasome is the most well-characterized, it can be activated by many stimuli, including adenosine triphosphate (ATP), nigericin, monocrystalline sodium urate (MSU), SiO_2_, cholesterol crystals and amyloid-β aggregates^17–19^. Thus, the NLRP3 inflammasome contributes to the development of several human diseases, including gout, Alzheimer’s disease, enteritis and liver disease^4,8,20,21^. In recent years, several molecular compounds, including MCC950, OLT1177, Bay 11-7082, β-hydroxybutyrate glyburide, parthenolide, sulforaphane, glycyclamide, isoliquiritigenin and tranilast^22–25^, have been shown to have clear inhibitory effect on the NLRP3 inflammasome. MCC950, the most potent and specific inhibitor of NLRP3, has proven efficacy in many mouse models of NLRP3-driven diseases, such as colitis, NASH, Alzheimer’s disease and other afflictions^26–29^. However, its potential hepatotoxicity has been confirmed in phase II clinical trials^30^. Aside from MCC950, only OLT1177 has been tested in phase II clinical trials^13,25^. It is, therefore, urgent to develop safe and effective NLRP3 inflammasome inhibitors for the treatment of inflammasome-mediated diseases.

Heat-shock protein 90 (HSP90), a molecular chaperone, modulates the stability and activation of other proteins (clients) involved in protein trafficking, signal transduction and receptor maturation^31–33^. Moreover, HSP90 is responsible for stabilizing NLR proteins, such as NLRP3^34^. HSP90 is also essential for the activation of NLRP3 inflammasome, and geldanamycin(GA), a specific inhibitor of HSP90, blocks NLRP3 inflammasome activation and helps ameliorate NLRP3 inflammasome-mediated diseases^35^.

Herbal rosemary and sage have been widely used around the world for both culinary purposes and their medicinal properties^36^. Rosemary and sage have both been shown to contain a variety of polyphenols, including carnosol and carnosic acid^37^. Polyphenols extracted from rosemary exhibit strong antioxidant activity, and carnosol and carnosic acid account for ~90% of this antioxidant activity^38,39^. Carnosol has previously been shown to exhibit anti-inflammatory activity and prevent the activation of various inflammatory signaling pathways, such as the NF-κB and mitogen-activated protein kinase (MAPK)^40^ pathways. Therefore, carnosol is considered to be promising anti-inflammatory agent.

In this study, we demonstrate that carnosol treatment inhibits NLRP3 inflammasome activation by directly interacting with HSP90 and blocking its ATPase activity. More importantly, carnosol treatment prevents or alleviates NLRP3 inflammasome-mediated diseases in mouse models, indicating that carnosol is a potential candidate for the treatment of inflammasome-mediated human diseases.

## Results

### Carnosol inhibits NLRP3 inflammasome activation in BMDMs, THP1 cells and hPBMCs

**Fig. 1:**
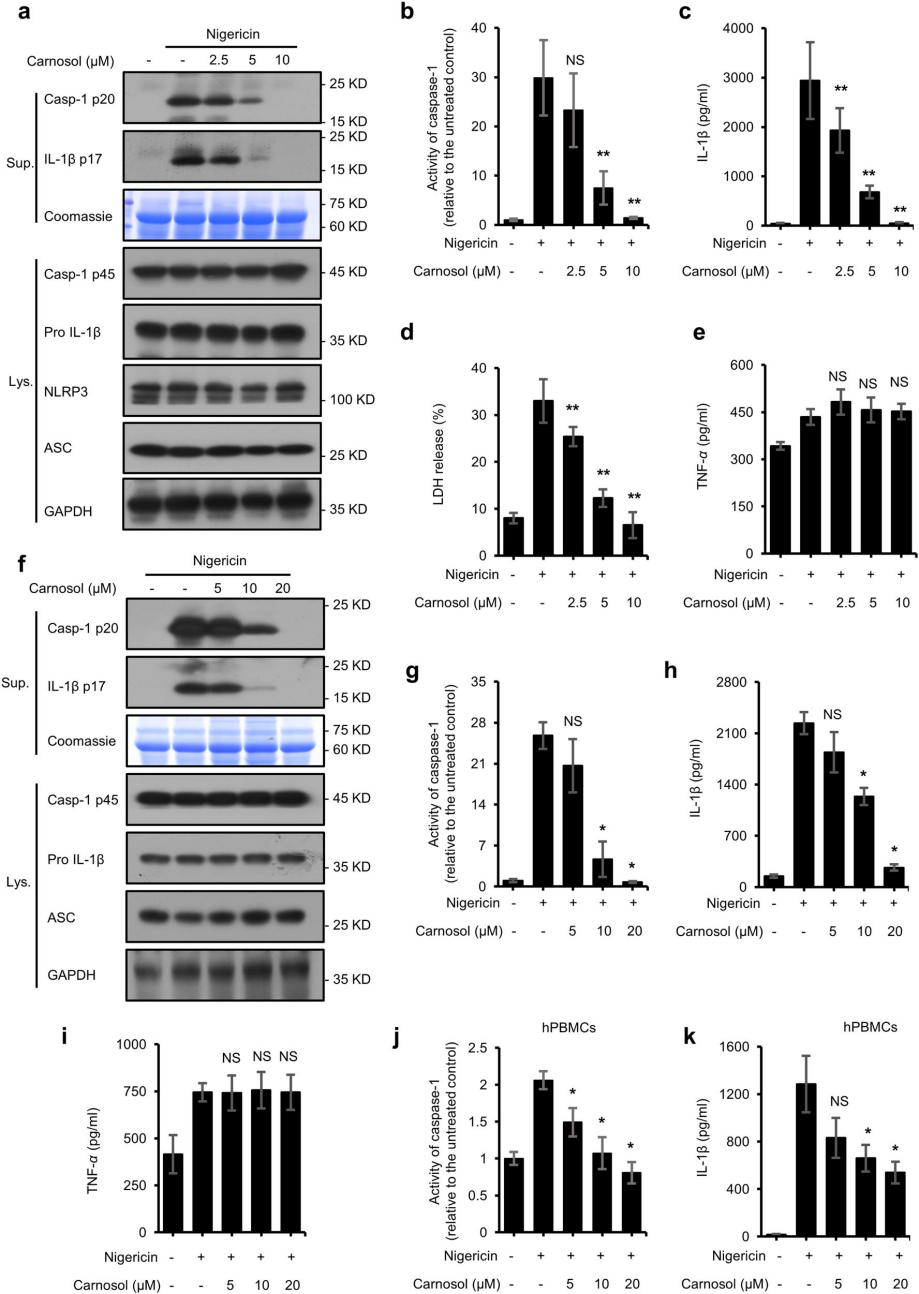
Carnosol inhibits NLRP3 inflammasome activation triggered by nigericin in BMDMs, THP1, and PBMCs. **a** Western blot analysis of caspase-1 (p20) and IL-1β in culture supernatants (Sup.) and pro- IL-1β, caspase-1 (p45), NLRP3 and ASC in cell lysates (Lys.) of LPS-primed BMDMs treated with various doses of carnosol and then stimulated with nigericin. **b**–**e** Activity of caspase-1 (**b**), ELISA of IL-1β (**c**), release of LDH (**d**) and ELISA of TNF-α (**e**) in Sup. from samples described in **a**. **f** Western blot analysis of caspase-1 (p20) and IL-1β in Sup. and pro- IL-1β, caspase-1 (p45), NLRP3 and ASC in cell Lys. of PMA-primed THP1 treated with various doses of carnosol and then stimulated with nigericin. **g**–**i** Activity of caspase-1 (**g**), ELISA of IL-1β (**h**) and TNF-α (**i**) in Sup. from samples described in **f**. **j**, **k** Activity of caspase-1 (**j**) and ELISA of IL-1β (**k)** in Sup. from LPS-primed hPBMCs treated with various doses of carnosol and then stimulated with nigericin. Coomassie blue staining was used as the loading control in Sup. GAPDH served as a loading control in the Lys. Data are represented as the mean ± SD from at least four biological samples. The significance of the differences was analyzed using Mann–Whitney *U* test: *P < 0.05, **P < 0.01, ***P < 0.001 vs. the control, NS, not significant.

A high-throughput assay for bioluminescent caspase-1 activity in screening NLRP3 inflammasome inhibitors revealed that carnosol inhibits NLRP3 inflammasome activation (data not shown). To further investigate how carnosol impacts NLRP3 inflammasome activation, we pretreated LPS-primed BMDMs with carnosol prior to nigericin stimulation. Our results showed that carnosol inhibited caspase-1 and IL-1β production in a dose-dependent manner, as well as the release of LDH in LPS-primed BMDMs (Fig. 1a–d). Similarly, pretreatment with carnosol also dose-dependently inhibited the nigericin-induced maturation of caspase-1 and IL-1β in PMA-primed THP1 cells (Fig. 1f–h). Furthermore, carnosol suppressed nigericin-induced caspase-1 activation and IL-1β maturation in LPS-primed hPBMCs (Fig. 1j, k). These results suggest that carnosol inhibits nigericin-mediated NLRP3 inflammasome activation. In contrast to the secretion of IL-1β, the inflammasome-independent secretion of tumor necrosis factor-α (TNF-α) and the expression of NLRP3 inflammasome complex proteins, including NLRP3, ASC, procaspase-1 and pro-IL-1β, were not affected by carnosol treatment (Fig. 1a, e, f, i), suggesting that carnosol affects the activation of NLRP3 inflammasome.

**Fig. 2:**
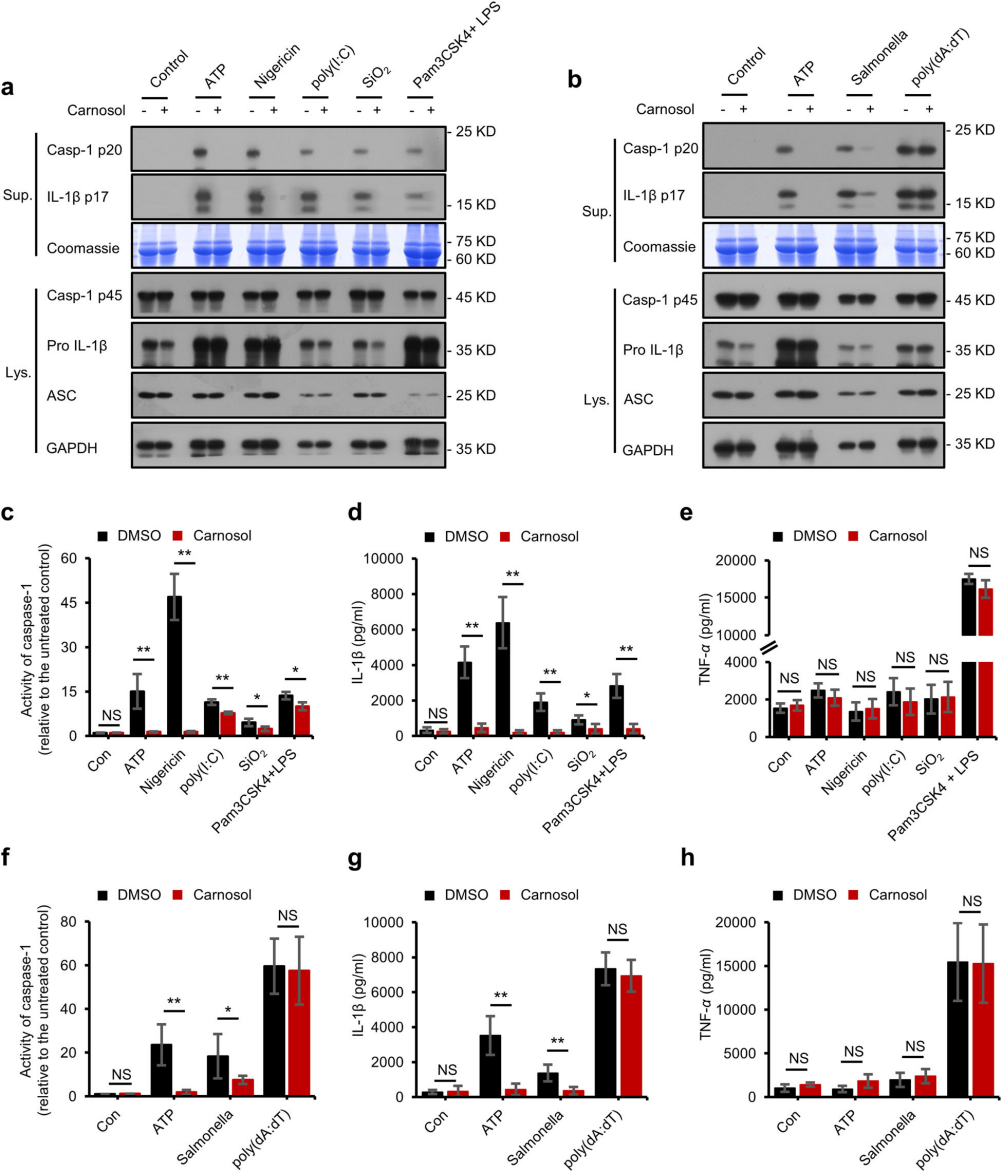
Carnosol inhibits other stimuli-induced NLRP3 inflammasome activation and also inhibits NLRC4 inflammasome activation. **a**, **b** Western blot analysis of caspase-1 (p20) and IL-1β in supernatants (Sup.) of LPS-primed BMDMs treated with carnosol (10 μM) and then stimulated with ATP, nigericin, poly(I:C), SiO_2_, poly(dA:dT) and *Salmonella* or of Pam3CSK4-primed BMDMs treated with carnosol (10 μM) and then stimulated with LPS and Western blot analysis of pro- IL-1β, caspase-1 (p45), NLRP3, and ASC in cell lysates (Lys.). **c–h** Activity of caspase-1 (**c**, **f**), ELISA of IL-1β and TNF-α (**d**, **e**, **g**, **h**) in Sup. from samples described in **a**,**b**. Coomassie blue staining was used as the loading control in the Sup. GAPDH served as a loading control in the Lys. Data are represented as the mean ± SD from at least four biological samples. The significance of the differences was analyzed using Mann–Whitney *U* test: *P < 0.05, **P < 0.01, ***P < 0.001 vs. the control, NS: not significant.

To determine whether carnosol acts as a broad-spectrum inhibitor of the NLRP3 inflammasome, we investigated the effect of carnosol on the SiO2-, poly(I:C)- and cytosolic LPS-mediated activation of the NLRP3 inflammasome. We observed that carnosol treatment disrupted caspase-1 cleavage and IL-1β maturation, which were triggered by these NLRP3 inflammasome stimuli (Fig. 2a, c, d). Meanwhile, carnosol treatment had no effect on the production of TNF-α and NLRP3 inflammasome complex proteins (Fig. 2a, e). Taken together, these results demonstrate that carnosol treatment inhibits the activation of the NLRP3 inflammasome.

### Carnosol inhibits NLRC4 inflammasome activation but has no effect on AIM2 inflammasome activation and NF-κB-mediated induction of inflammasome complex proteins

Next, we tested whether the inhibitory effect of carnosol on NLRP3 inflammasome was specific. The NLRC4 inflammasome can be activated by flagellin derived from bacteria, such as *Salmonella typhimurium*^15,41–43^. We evaluated whether carnosol treatment could prevent NLRC4 inflammasome activation, and our results showed that carnosol treatment disrupted NLRC4-dependent caspase-1 activation as well as IL-1β secretion in *Salmonella*-infected LPS-primed BMDMs (Fig. 2b, f, g), whereas TNF-α production remained unchanged (Fig. 2h). Furthermore, the expression of NLRC4 inflammasome complex proteins, including procaspase-1, pro-IL-1β and ASC, were not impaired by carnosol treatment (Fig. 2b). The AIM2 inflammasome can be activated by double-stranded DNA and induces inflammation^44–47^. We observed that carnosol had no effect on caspase-1 maturation or IL-1β and TNF-α expression in LPS-primed BMDMs after poly(dA:dT) transfection (Fig. 2b, f–h). These results revealed that carnosol inhibits NLRC4 inflammasome activation but has no effect on AIM2 inflammasome activation.

Previous studies have shown that carnosol disrupts the activation of the NF-κB signaling pathway, which largely controls NLRs and pro-IL-1β expression^40,48^. Our study revealed that when BMDMs were first treated with carnosol for 1 h and then stimulated with LPS for 4 h, carnosol treatment inhibited the expression of pro-IL-1β, TNF-α, and IL-6 in BMDMs (Fig. S1a–c). However, when BMDMs were first stimulated with LPS for 4 h and then treated with carnosol for 1 h, the expression of NLRP3, pro-IL-1β, TNF-α and IL-6 was not affected (Fig. S1a–c). These findings imply that the inhibitory effect of carnosol on the activation of inflammasomes is not related to the NF-κB-mediated expression of NLRP3 and pro-IL-1β.

### Carnosol inhibits the assembly of inflammasome complexes but has no effect on mitochondrial damage

**Fig. 3:**
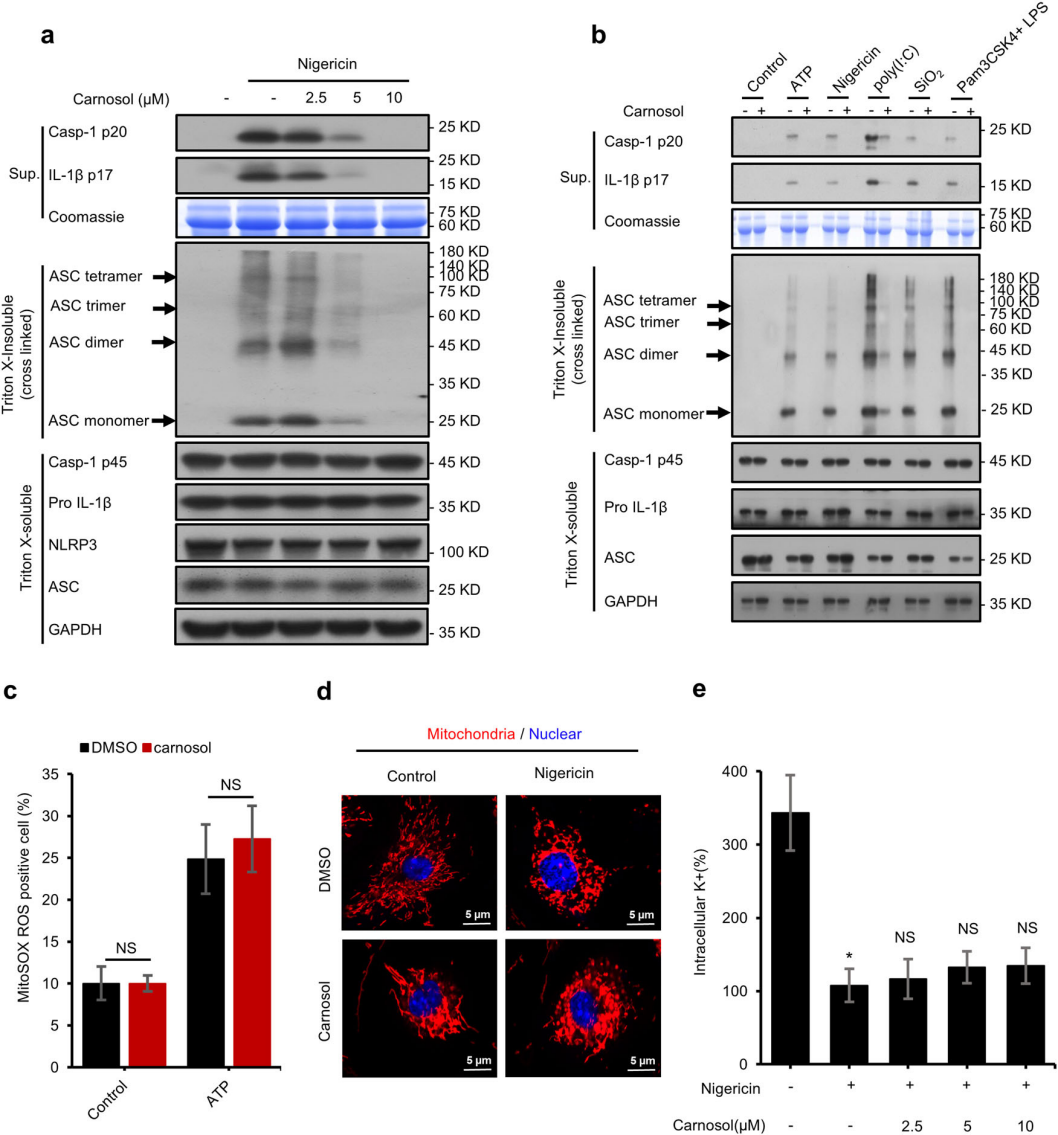
Carnosol inhibits the assembly of inflammasome complexes but has no effect on mitochondrial damage. **a** Western blot analysis of ASC oligomerization in cell lysates of LPS-primed BMDMs treated with various doses of carnosol and then stimulated with nigericin. **b** Western blot analysis of ASC oligomerization in cell lysates of LPS-primed BMDMs treated with carnosol (10 μM) and then stimulated with ATP, nigericin, poly(I:C), SiO_2_ or in cell lysates of Pam3CSK4-primed BMDMs treated with carnosol (10 μM) and then stimulated with LPS. **c** Percentage of ROS-positive cells in LPS-primed BMDMs with carnosol and then stimulated with or without ATP, followed by staining with MitoSox. **d** Confocal microscopy analysis in LPS-primed BMDMs treated with carnosol and then stimulated with nigericin for 30 min, followed by staining with MitoTracker Red. Scale bars represent 5 µm. **e** Qualification of potassium efflux in LPS-primed BMDMs treated with various doses of carnosol and then stimulated with nigericin. Data are represented as the mean ± SD from at least four biological samples. The significance of the differences was analyzed using Mann–Whitney U test: *P < 0.05, **P < 0.01, ***P < 0.001 vs. the control, NS: not significant.

NLRP3 requires ASC for the recruitment of procaspase-1 to form inflammasome complexes^46,48,49^. We further assessed the effect of carnosol on the formation of ASC oligomers, which is an important step in the activation of NLRP3 inflammasome^17,48^. Consistent with the inhibitory effects of carnosol on caspase-1 activation and IL-1β production, carnosol treatment also dose-dependently blocked ASC oligomerization induced by nigericin in LPS-primed BMDMs and PMA-primed THP1 cells (Figs. 3a, S2). In addition, carnosol treatment also inhibited NLRP3-dependent ASC oligomerization triggered by ATP, poly(I:C), SiO_2_ and cytosolic LPS (Fig. 3b). These results suggest that carnosol may directly target ASC oligomerization or upstream events to block NLRP3 inflammasome activation.

Oxidative stress is one of the most important events upstream of inflammasome activation^48–50^. Therefore, we examined the effect of carnosol on the production of reactive oxygen species (ROS) induced by ATP. Our results revealed that carnosol treatment had no effect on the release of ROS induced by ATP (Fig. 3c). Previous studies have revealed that mitochondrial damage is associated with activation of NLRP3 inflammasome^49,51^. Thus, we utilized MitoTracker Red to stain mitochondria in BMDMs that were pretreated with carnosol and/or nigericin. Our results revealed that carnosol treatment did not inhibit nigericin-induced mitochondrial damage (Fig. 3d). These findings collectively suggest that carnosol has no effect on mitochondrial damage during inflammasome activation. Potassium efflux is another important upstream signaling pathway of NLRP3 inflammasome activation^52,53^. Nigericin treatment can cause a dramatic decrease in intracellular potassium, but this effect was not suppressed by carnosol (Fig. 3e), suggesting that carnosol has no effect on potassium efflux during NLRP3 inflammasome activation.

### Carnosol inhibits inflammasome assembly by directly targeting HSP90 and inhibiting its ATPase activity

**Fig. 4:**
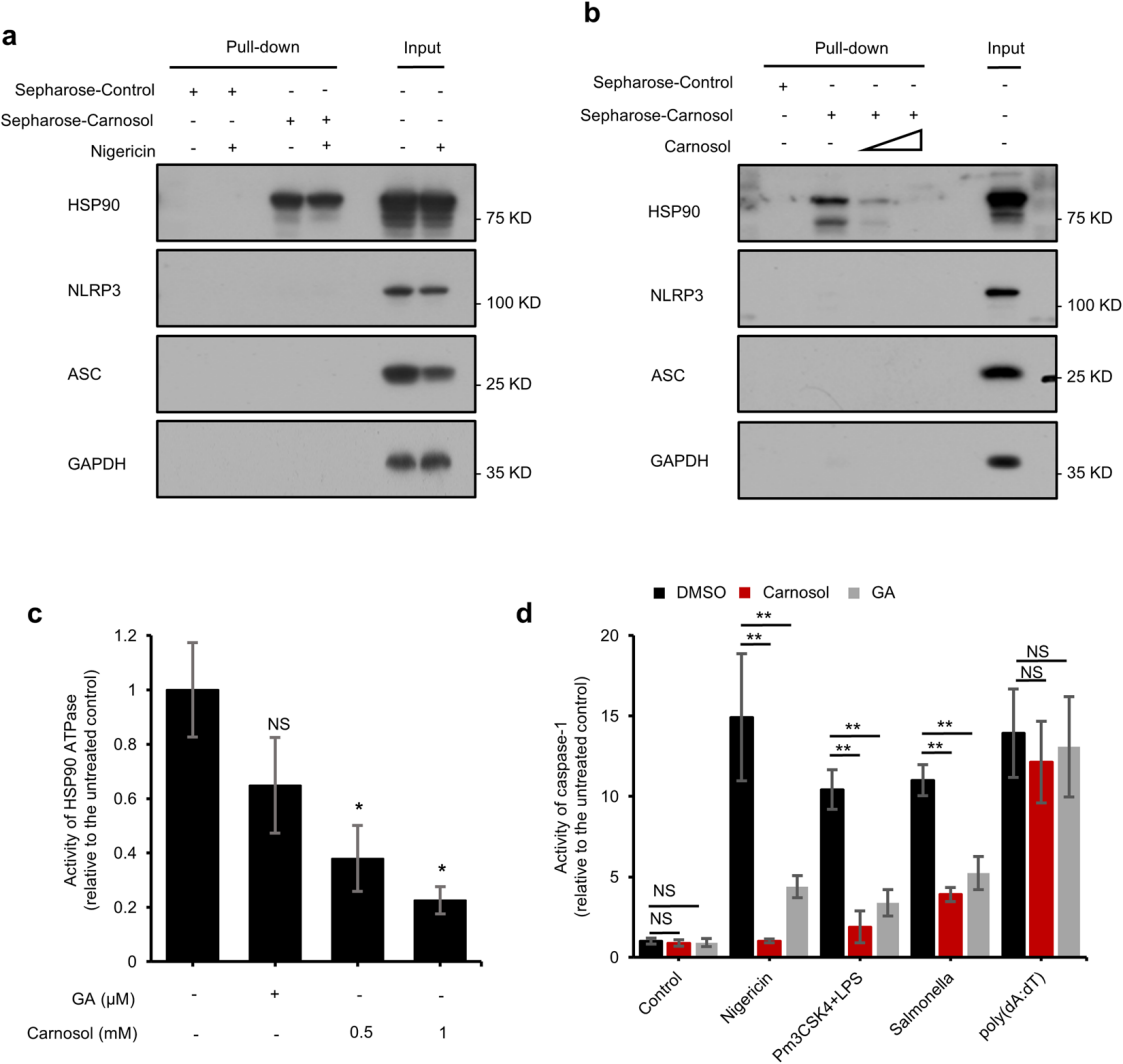
Carnosol inhibits inflammasome activation by directly targeting HSP90 and then inhibits its ATPase activity. **a** Cell lysates of LPS-primed BMDMs stimulated with or without nigericin. Cells were incubated with sepharose-carnosol for 12 h and proteins pulled down with sepharose beads. **b** Cell lysates of LPS-primed BMDMs. Cells were incubated with sepharose-carnosol and carnosol for 12 h and proteins pulled down with sepharose beads. **c** Effect of carnosol on the Activity of HSP90 ATPase. After incubation with HSP90 plus DMSO, HSP90 plus carnosol (0.5 mM, 1 mM), or HSP90 plus GA (30 μM), ATP was measured by Cell Titer Glo. **d** Activity of caspase-1 in Sup. from LPS-primed BMDMs treated with carnosol (10 μM) or GA (10 μM) and then stimulated with nigericin, poly(dA:dT) and *Salmonella* or from Pam3CSK4-primed BMDMs treated with carnosol (10 μM) or GA (10 μM) and then stimulated with LPS. Data are represented as the mean ± SD from at least four biological samples. The significance of the differences was analyzed using Mann–Whitney *U* test: *P < 0.05, **P < 0.01, ***P < 0.001 vs. the control, NS: not significant.

To further elucidate the inhibitory mechanism of carnosol on inflammasome activation, we synthesized carnosol with cyanogen bromide-activated Sepharose (Sepharose-carnosol) and investigated whether carnosol could directly bind to the proteins involved in NLRP3 inflammasome. We found that HSP90, but not ASC or NLRP3, was pulled down by the Sepharose-carnosol (Fig. 4a). It has been reported that HSP90 is essential for the activation of the NLRP3 inflammasome^32,54^. In order to exclude the possibility of nonspecific binding of Sepharose-carnosol to HSP90, we next incubated cell lysates with free carnosol and then added Sepharose-carnosol. The results showed that free carnosol dose-dependently inhibited the binding of Sepharose-carnosol to HSP90 (Fig. 4b), confirming that carnosol can indeed directly interact with HSP90.

The ATPase activity of HSP90 constitutes a major role in the control of inflammasome activation, as indicated by treatment using HSP90 inhibitors such as GA and 17-DMAG^55^. We further examined whether carnosol-induced inhibition of inflammasome activation involves the inhibition of HSP90 ATPase activity. Our results showed that carnosol treatment dose-dependently inhibited the ATPase activity of HSP90 in vitro (Fig. 4c). Similar to the findings of previous reports, GA treatment inhibited caspase-1 activation, IL-1β maturation and ASC oligomerization triggered by nigericin, cytosolic LPS or *Salmonella* infection, aside from poly(dA:dT) transfection (Fig. 4d). These results suggested that carnosol blocks the activation of the NLRP3 and NLRC4 inflammasomes by binding to HSP90 and inhibiting its ATPase activity.

### Carnosol prevents NLRP3 inflammasome activation and LPS-induced septic shock in mice

**Fig. 5:**
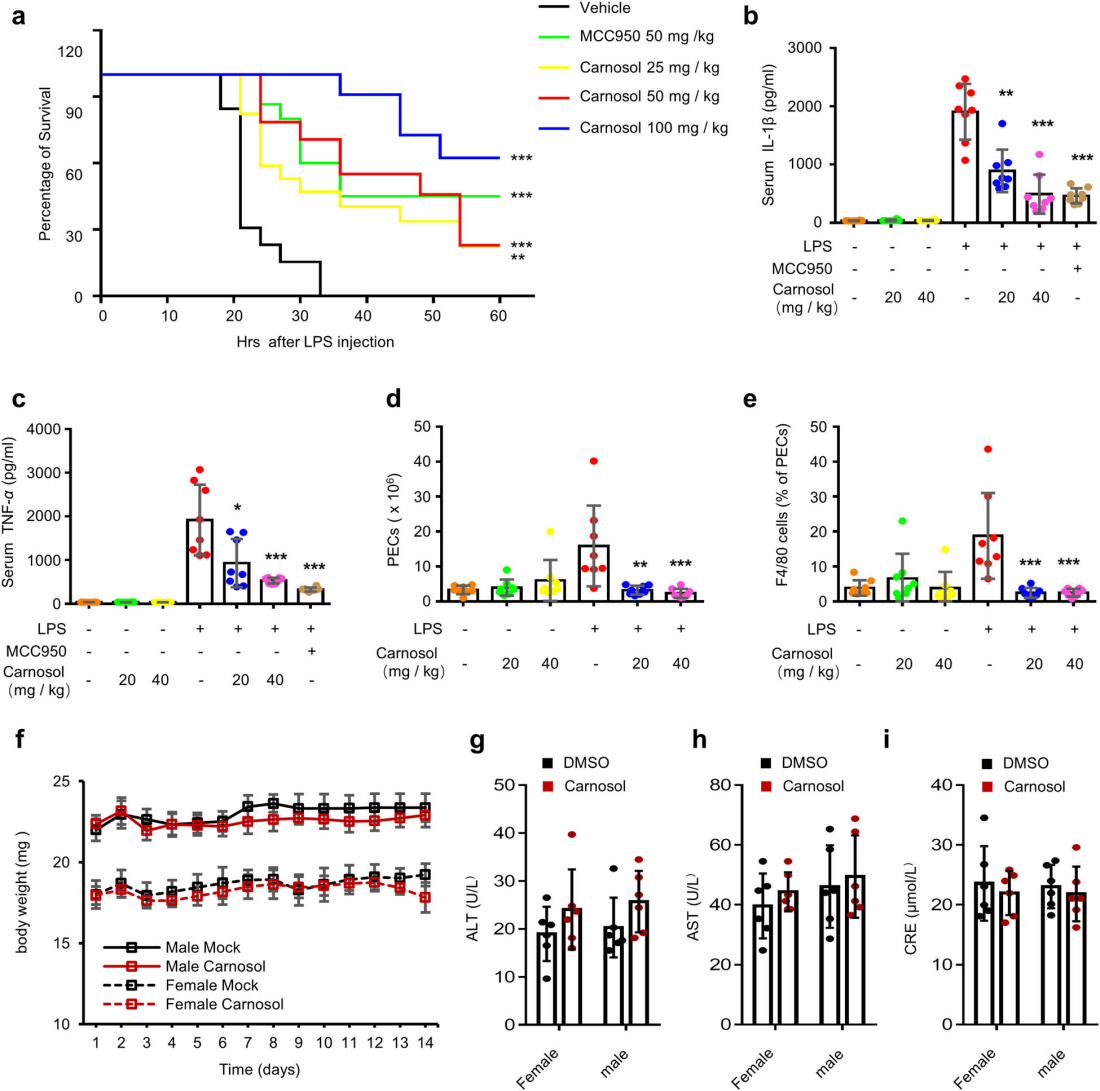
Carnosol prevents NLRP3 inflammasome activation and suppresses LPS-induced septic shock in mice. **a** Survival of C57BL/6 female mice treated with vehicle, MCC950 (50 mg/kg) or various doses of carnosol and then intraperitoneally injected with LPS (20 mg/kg). Survival was monitored for 60 h (n = 10). **b**–**e** ELISA of serum IL-1β (**b**), TNF-α (**c**) and quantification of peritoneal exudate cells (PECs) (**d**), monocytes-macrophages (F4/80^+^ cells) (**e**) from C57BL/6 female mice treated with vehicle, various doses of carnosol or MCC950 (40 mg/kg) for 1 h and then intraperitoneally injected with LPS (20 mg/kg) for 4 h. **f**–**i** Changes in body weight (**f**), ALT (**g**), AST (**h**) and CRE (**i**) levels in C57BL/6 mice treated with vehicle or carnosol (120 mg/kg) for 14 days. Data are represented as the mean ± SD. The significance of the differences was analyzed using Mann–Whitney *U* test or log-rank test: *P < 0.05, **P < 0.01, ***P < 0.001 vs. the control, NS, not significant.

To test whether carnosol inhibits NLRP3 inflammasome activation in vivo, we chose the NLRP3 inflammasome-dependent septic shock mouse model induced by intraperitoneal injection of LPS^56,57^. Mice were intraperitoneally injected with MCC950 or carnosol for 1 h before being injected with LPS and were then monitored for survival. Our results showed that carnosol treatment dose-dependently improved the survival of mice with LPS-induced septic shock (Fig. 5a). We also compared the effect of carnosol with that of MCC950, which is considered to be a selective inhibitor of the NLRP3 inflammasome^22^, and the found that the protective effect of carnosol against LPS-mediated lethality was similar to that of MCC950 (Fig. 5a). Additionally, mice were initially injected with carnosol or MCC950 intraperitoneally and then injected with LPS 1 h later, followed by evaluation of NLRP3 inflammasome activation after 4 h. The results indicated that, similar to the effect of MCC950, treatment with carnosol downregulated IL-1β and TNF-α in the LPS-mediated septic shock mouse model in a dose-dependent manner, along with a reduction in the number of peritoneal exudate cells and peritoneal macrophages (Figs. 5b–e; S3a, b). Taken together, these results showed that carnosol treatment disrupts the activation of NLRP3 inflammasome and NLRP3-related septic shock in mice.

We then evaluated the toxicity effect of carnosol in vivo. Mice were injected with 120 mg/kg of carnosol intraperitoneally for two weeks, and it was found that carnosol did not induce changes in body weight or liver and kidney function in the mice (Fig. 5f–i), indicating that carnosol is well tolerated in mice and thus may be safe for use in the treatment of inflammasome-related diseases.

### Carnosol suppresses liver injury and fibrosis in an experimental NASH model

**Fig. 6:**
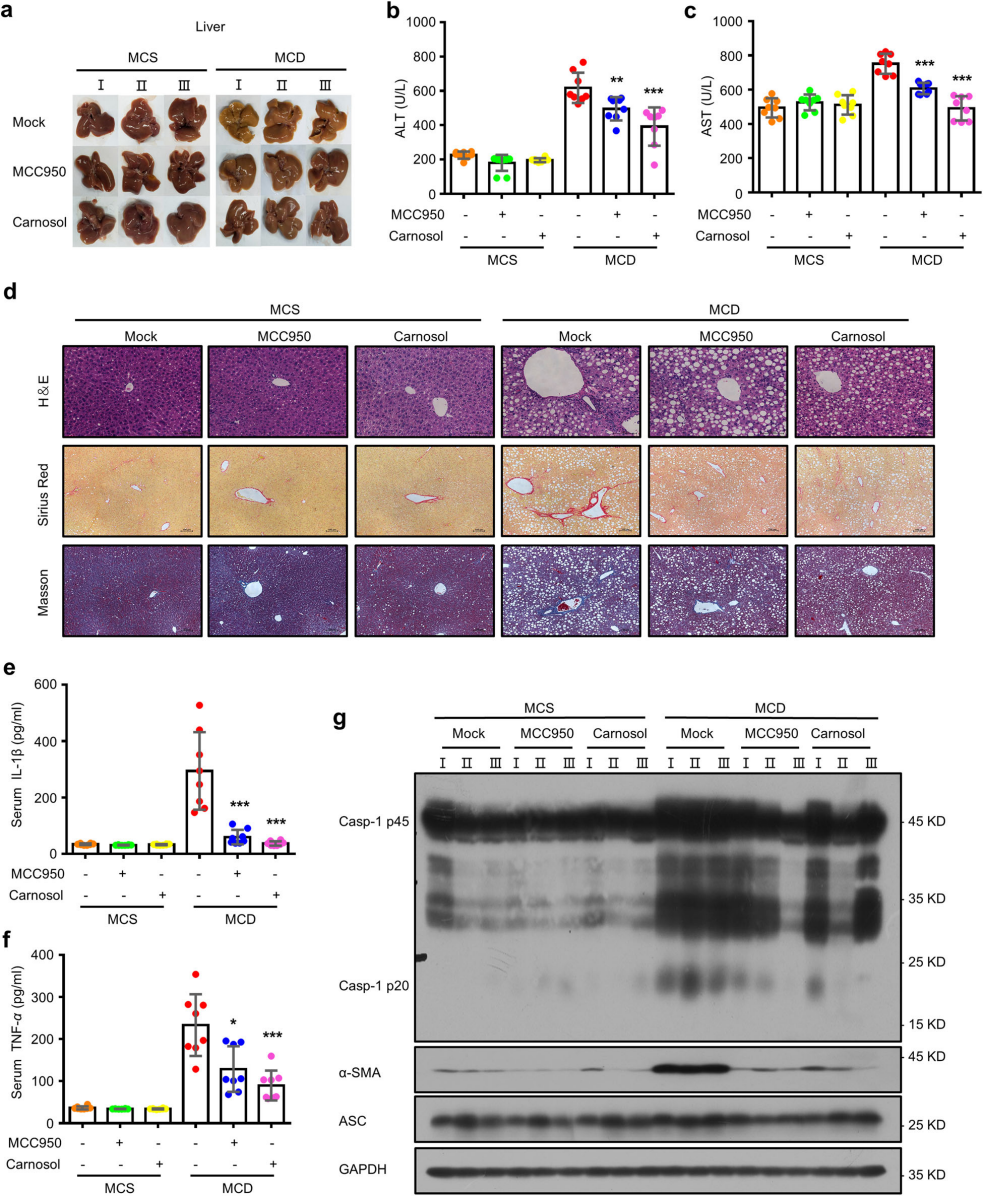
Carnosol suppresses liver injury and fibrosis in an experimental NASH model. **a**–**h** Eight-week-old male C57BL/6 mice were placed on a methionine- and choline-deficient (MCD) diet or an identical diet supplemented with methionine and choline (MCS) for 6 weeks. The mice were treated with carnosol (20 mg/kg) or MCC950 (20 mg/kg) every day for 5 days and then treated with carnosol (40 mg/kg) or MCC950 (40 mg/kg) every 2 days for up to 6 weeks. **a** Macroscopic appearance of the liver. **b**, **c** Serum levels of ALT (**b**) and AST (**c**). **d** Representative micrographs of liver H&E staining, Masson staining, and Sirius red staining. Scale bars represent 100 µm. **e**, **f** ELISA of IL-1β (**e**) and TNF-α (**f**). **g** The levels of pro caspase-1, cleaved caspase-1, α-SMA and ASC proteins in the liver were determined by western blotting. Data are represented as the mean ± SD. The significance of the differences was analyzed using Mann–Whitney *U* test: *P < 0.05, **P < 0.01, ***P < 0.001 vs. the control, NS, not significant.

Since the NLRP3 inflammasome is mechanistically important for the development of NASH^58^, we next evaluated the effects of carnosol in a methionine- and choline-deficient (MCD) diet-fed mouse model of NASH. We observed that, relative to methionine- and choline-sufficient (MCS) diet-fed mice, major changes in liver morphology, which were reversed by carnosol treatment, were observed in MCD diet-fed mice (Fig. 6a). Furthermore, compared to the MCS diet-fed mice, we observed higher plasma alanine aminotransferase (ALT) and aspartate aminotransferase (AST) levels in the MCD diet-fed mice, and these symptoms were prevented by carnosol treatment (Fig. 6b, c). In addition, liver histopathological analysis revealed fat vacuoles, cell death and inflammatory cell infiltration in the livers of MCD diet-fed mice, and this hepatic fibrosis was noticeably relieved by carnosol treatment, as evidenced by Masson staining and Sirius red staining (Fig. 6d). MCC950, an NLRP3 inflammasome inhibitor, has been shown to be useful for treating NASH in MCD diet-fed mice^27^. As expected, treatment with MCC950 resulted in an improvement in NASH pathology and liver fibrosis in the MCD diet-fed mice, and the inhibitory effect of carnosol was comparable to that of MCC950 (Fig. 6a–d).

To validate our findings that carnosol treatment ameliorated NASH by suppressing NLRP3 inflammasome activation, we assessed the activation of the NLRP3 inflammasome using a NASH model. We determined that treatment with carnosol or MCC950 resulted in a decrease in IL-1β, TNF-α and profibrotic marker alpha-smooth muscle actin (α-SMA) expression in MCD diet-fed mice (Figs. 6e–g; S3c, d). In addition, the increase in cleaved caspase-1 in MCD diet-fed mice was also suppressed by carnosol or MCC950 treatment (Fig. 6g). Thus, these findings suggest that carnosol improves NASH by disrupting NLRP3 inflammasome activation.

## Discussion

In this study, we demonstrated that carnosol has a strong inhibitory effect on NLRP3 inflammasomes. Our findings showed that carnosol inhibits inflammasomes by binding to HSP90 and then inhibiting its ATPase activity, which is essential for NLRP3 inflammasome activation. We also demonstrated that carnosol can prevent or treat NLRP3 inflammasome-driven human diseases, including septic shock and NASH in mouse models. In addition, we also confirmed that intraperitoneal administration of carnosol (120 mg/kg) once daily for 2 weeks is well tolerated in mice. Thus, our study suggests that carnosol is a safe and effective candidate for the treatment of NLRP3-driven diseases.

Previous studies have demonstrated that carnosol targets the NF-κB signaling pathway^40,59,60^, and our data showed that carnosol indeed inhibited NF-κB-mediated pro-IL-1β expression and IL-6 production in BMDMs treated with carnosol before LPS stimulating, indicating it could also prevent the inflammasome priming if added prior to the priming signal. Moreover, carnosol had no effect on expression of pro-IL-1β and IL-6 in BMDMs treated with carnosol after LPS stimulating, suggesting that carnosol can inhibit the priming stage but also plays a role in NLRP3 inflammasome activation step.

To further clarify the target of the carnosol-mediated inhibition of inflammasomes activation, we investigated the interaction between carnosol with inflammasome-related proteins. Our results demonstrated that carnosol can directly interact with HSP90, which is essential for NLRP3-inflammasome activation^35^. We also found that carnosol treatment inhibited the ATPase activity of HSP90, which is necessary for NLRP3 and NLRC4 inflammasome activation, as shown by 17-DMAG and GA, inhibitors of the ATPase activity of HSP90^31,33,55^. Consistent with the effect of GA, our results showed that carnosol also blocked NLRP3 and NLRC4, but not AIM2 inflammasome activation. Our results also showed that carnosol had no effect on ASC oligomerization transfected by poly(dA:dT) and subsequent AIM2 inflammasome activation and similar results were obtained in the GA group. Thus, these results demonstrate that carnosol treatment inhibits NLRP3 and NLRC4 inflammasome activation by blocking HSP90 and disrupting its ATPase activity.

Previous studies have shown that the inflammasome components become secreted out of the cell together upon inflammasome activation^61–63^, on the other hand, recent studies show that the levels of inflammasome components like ASC, caspase-1, and NLRP3 remain unchanged in the cell lysate^24,64–66^. The contradiction may be due to the difference in the cell type, stimuli and the stimulation time or strength. In most of our experiments, the expression of NLRP3 inflammasome complex proteins were not affected by carnosol treatment, that’s may be because of the weak stimuli and the short time of stimulation in our study.

Our results also suggest that carnosol has definite therapeutic effects in mouse models of various NLRP3 inflammasome-mediated diseases, including septic shock and NASH. Although the initial effective dose of carnosol needed to inhibit NLRP3 inflammasome activation was higher than that of MCC950 in vitro, the rescue effect of carnosol on inflammasome-related diseases was comparable to that of MCC950 in all tested animal models of human diseases, suggesting that carnosol has a therapeutic potential equivalent to that of MCC950 for inflammasome-mediated diseases.

Many investigations have provided evidence that carnosol is well tolerated in short- and long-term toxicity experiments^67–69^. Previous studies also suggested that daily intraperitoneal administration of carnosol is well tolerated^70,71^. As for the application route, it has been shown that the most common application route of carnosol is i.p. injection and lots of other molecules, such as MCC950 and Formononetin, are also used in this application route^22,70–73^.Intraperitoneal administration of carnosol with 200 mg/kg daily for 5 days has no effect on liver weight^73^. Furthermore, we confirmed that carnosol is well tolerated in mice when administered intraperitoneally at 120 mg/kg daily for 2 weeks. It has also been reported that carnosol alleviates the colorectal cancer risk, when used as an additive to cured meat, and ultraviolet-induced erythema in the clinical trials^74,75^, suggesting that oral administration may an appropriate application route in humans. However, the bioavailability and metabolism of carnosol remains to be further studied. Thus, considering its protective effect and safety, carnosol is a strong candidate for the treatment of inflammasome-mediated diseases, but additional studies are needed to determine its therapeutic effects in humans.

## Materials and methods

### Mice

Eight-week-old C57BL/6 mice were obtained from SPF Biotechnology Co., Ltd. (Beijing, China). The animals were allowed unlimited access to food and water for the entire experiment except during fasting assays and were kept under a 12-h light/dark cycle. The animal experiments were conducted according to the guidelines for the care and use of laboratory animals and were approved by the Fifth Medical Center of PLA General Hospital, Beijing, China. We tried our best to minimize both the suffering and the number of animals used. When assessing experimental outcomes, the investigators were blinded to the treatments.

### Cell culture

BMDMs were collected from the bone marrow of 10-week-old female mice and then cultured for 6–7 days in Dulbecco’s modified Eagle’s medium (DMEM) containing 10% fetal bovine serum (FBS), 1% penicillin/streptomycin (P/S) and 50 ng/mL murine macrophage colony-stimulating factor (M-CSF). Human THP-1 cells and human PBMCs were cultured in RPMI 1640 medium and all of the culture media were supplemented with 10% FBS and 1% penicillin/streptomycin (P/S). Cells were kept in a humidified 5% (v/v) CO_2_ incubator at 37 °C. Human THP-1 cells were a gift from Dr. Tao Li of the National Center of Biomedical Analysis.

### Antibodies and reagents

Nigericin, ATP, poly(dA:dT), poly(I:C), phorbol-12-myristate-13-acetate (PMA), dimethyl sulfoxide (DMSO) and ultrapure LPS were obtained from Sigma-Aldrich (Munich, Germany). Silicon dioxide (SiO_2_) and Pam3CSK4 were obtained from InvivoGen (Toulouse, France). MCC950, carnosol, and geldanamycin (GA) were obtained from TargetMol (Boston, MA, USA). MitoTracker and MitoSOX were manufactured by Invitrogen (Carlsbad, CA, USA). Salmonella was a gift from Dr. Tao Li of the National Center of Biomedical Analysis. Anti-mouse Caspase-1 (1:1000, AG-20B-0042) was from Adipogen (San Diego, USA). Anti-human cleaved IL-1β (1:2000, 12242), Anti-mouse α-SMA (1:1000, 19245s), anti-human Caspase-1(1:2000, 4199S), anti-mouse IL-1β (1:1000, 12507) and anti-NLRP3 (1:2000, 15101S) were from Cell Signaling Technology (Boston, USA). Anti-ASC (1:1000, sc-22514-R) was from Santa Cruz Biotechnology (Dallas, USA). Anti-DDDK tag (1:3000, 20543–1-AP), Anti-HSP90 (1:3000, 13171-1-AP) and Anti-GAPDH (1:2000, 60004-1-1G) were from Proteintech Group (Chicago, USA).

### Human samples

Adult peripheral blood samples were collected from three healthy donors following informed consent, and all experimental protocols were conducted following the guidelines of the Institutional Human Research Subjects Protection Committee of the Ethics Committee of the Fifth Medical Center of Chinese PLA General Hospital.

### Inflammasome activation

BMDMs, THP-1 cells, and PBMCs were seeded into 24-well plates at a density 5 × 10^5^ cells/well, 7.5 × 10^5^ cells/well and 2.5 × 10^6^ cells/well. After 12–18 h, we replaced the culture medium with fresh media and cell priming was performed using LPS (50 ng/mL), PMA (100 nmol/L), or Pam3CSK4 (1 μg/mL) for 4 h. Then, the cells were exposed to carnosol in Opti-MEM for 30 min. Inflammasome activation was performed as described previously^76^.

### Western blotting

Protein extraction of cell culture supernatants and western blotting assays were performed as described previously^76^.

### Caspase-1 activity assay

A Caspase-Glo^®^ 1 Inflammasome Assay (Promega, Madison, WI, USA) was employed to determine caspase-1 activity in cell culture supernatants following the manufacturer’s instructions.

### Enzyme-linked immunosorbent assay (ELISA)

Cell culture supernatants, mouse serum and tissue culture cells were assayed for mouse IL-1β (Cat: SMLB00C, R&D Systems, Minneapolis, MN, USA), TNF-α (Cat: 1217202, Dakewei, Beijing, China), IL-6 (Cat: 1210602, Dakewei, Beijing, China), human IL-1β (Cat: 1110122, Dakewei, Beijing, China) and TNF-α (Cat: 1117202, Dakewei, Beijing, China), according to the manufacturer’s instructions.

### Lactate dehydrogenase (LDH) assay

The release of LDH into the culture supernatants was assessed using a CytoTox 96^®^ 1 Non-radioactive Cytotoxicity Assay (Promega, Madison, WI, USA) following the manufacturer’s instructions.

### ASC oligomerization

The assay for ASC oligomerization was performed as described previously^77^.

### Confocal microscopy

Confocal microscopy was conducted as described previously^22^.

### ROS measurements

BMDMs were seeded at a density of 1 × 10^6^ cells/mL in 100-mm cell culture dishes. The next day, the medium was replaced and cell priming was performed using 50 ng/mL LPS for 4 h. The cells were then incubated in a test tube, and were later washed with Opti-MEM and stimulated as described earlier. The supernatants were discarded, and the cells were washed with EBSS, then stained with 4 μM MitoSOX for 20 min at 37 °C. The cells were then washed with EBSS, followed by flow cytometry. Data were acquired with an LSRFortessa Cell Analyzer (BD Biosciences, San Jose, CA, USA).

### Determination of intracellular potassium

BMDMs were plated overnight in 12-well plates and then primed with 50 ng/ml LPS for 4 h. After that, cells were treated with CS for 30 min and then stimulated with nigericin for 30 min. Culture medium was removed and cells were washed three times in potassium-free buffer (139 mM NaCl, 1.7 mM NaH_2_PO_4_, and 10 mM Na_2_HPO_4_, pH 7.2). 200 uL Ultrapure HNO_3_ was added to lyse the cells. Samples were transferred to glass bottles and then boiled for 30 min at 100 °C. After that, ddH_2_O was added to the samples for a total volume of 5 ml. Intracellular K+ measurements were performed by ICP-MS (Inductively coupled plasma mass spectrometry).

### Pull-down assay

Carnosol was conjugated with cyanogen bromide (CNBr)-activated Sepharose 4B (GE Healthcare). BMDMs were seeded at a density of 1 × 106 cells/mL overnight, followed by stimulation with or without nigericin. Then, BMDMs were lysed with a lysis buffer (25 mM Tris-HCl (pH 7.5), 0.5% Triton X-100, 150 mM NaCl, 0.5% sodium deoxycholate and 1% cocktail) and then centrifuged at 6000 × *g* for 20 min at 4 °C. Then, the supernatants were incubated with carnosol-conjugated Sepharose 4B at 4 °C overnight. Sepharose was prewashed thrice with coupling buffer. Carnosol was then mixed into the washed Sepharose and incubated for 24 h with constant rotation at 4 °C. The beads were washed thrice with lysis buffer. Then, the proteins that were pulled down were analyzed by immunoblotting.

### HSP90 ATPase assay

To assess the ATPase activity of HSP90, we incubated ATP with HSP90, DMSO, carnosol and GA for 1 h at 37 °C. To measure ATP levels, a CellTiter-Glo® Luminescent Cell Viability Assay kit (Promega, Madison, WI, USA) was used, following the manufacturer’s instructions.

### LPS-induced septic shock in vivo

Carnosol (25 mg/kg, 50 mg/kg or100 mg/kg) and MCC950 (50 mg/kg) were intraperitoneally (i.p.) injected into eight-week-old female C57BL/6 mice (*n* = 10/group). One hour later, the mice were injected with LPS (20 mg/kg). The mortality rate was monitored at regular intervals. In the second experiment, carnosol (20 mg/kg, 40 mg/kg) and MCC950 (40 mg/kg) were i.p. injected into eight-week-old female C57BL/6 mice (*n* = 8/group). One hour later, the mice were injected with LPS (20 mg/kg). After 4 h, we collected serum samples and peritoneal lavage fluids from the mice and cytokine levels were measured using ELISA.

### Toxicity of carnosol in vivo

Vehicle or carnosol (120 mg/kg/day) were injected into eight-week-old male or female C57BL/6 mice (*n* = 6/group). The body weights of the mice were measured daily for 14 days. At the end of the experiment, the mice were anesthetized and plasma samples were collected and assessed for AST, ALT and creatinine (CRE) levels according to the manufacturer’s instructions.

### Methionine- and choline-deficient diet model

Groups (*n* = 8/group) of eight-week-old male C57BL/6 mice were fed a methionine- and choline-deficient (MCD) diet (518810, Dyets, Bethlehem, PA, USA), whereas controls received an identical diet containing methionine and choline (MCS) (518811, Dyets). The MCD-fed mice and MCS-fed controls were separated into groups that received carnosol or MCC950 (20 mg/kg in 0.9% NaCl every day for a total of five days, and 40 mg/kg every second day, for up to six weeks) or vehicle by gavage. The mice were anesthetized at the end of the experiments and the liver and plasma were isolated.

### Statistical analysis

Statistical analysis was conducted using the GraphPad Prism 6 (GraphPad Software, San Diego, CA, US) and Microsoft Excel. The data are presented as the mean ± SD from at least four samples, the Mann–Whitney *U* test was used in our statistical analysis. Differences with a *P* value < 0.05 were deemed statistically significant. Statistical significance is presented as *P < 0.05, **P < 0.01, ***P < 0.001 vs. the control; NS, not significant.

## Supplementary information

Supplementary Figure Legends

Supplementary Figure 1

Supplementary Figure 2

Supplementary Figure 3
