# Supplementary material for: Carnosol inhibits inflammasome activation by directly targeting HSP90 to treat inflammasome-mediated diseases
Source: Cell Death Dis. 2020 Apr 20;11(4):252. doi: 10.1038/s41419-020-2460-x (PMC7170921; doi:10.1038/s41419-020-2460-x)
Supplement: Supplementary file 1 — Supplementary Figure Legends [file 41419_2020_2460_MOESM1_ESM.docx]

**Supplementary Figure Legends**

**Figure S1.** **Carnosol has no effect on NF-κB-mediated induction of inflammasome complex proteins a** Western blot analysis of the indicated proteins in Lys. from BMDMs treated with LPS for 4 h and stimulated with different doses of carnosol for 1 h (carnosol after LPS), or BMDMs treated with different doses of carnosol for 1 h and then stimulated with LPS for 4 h (carnosol before LPS). **b, c** ELISAs of TNF-α and IL-6 in Sup. from BMDMs described in **a**. Data are represented as the mean ± SD from at least four biological samples. The significant differences were analyzed using Mann-Whitney U test: *P < 0.05, **P < 0.01, ***P < 0.001 vs. the control, NS: not significant.

**Figure S2. Carnosol inhibits ASC oligomerization during NLRP3 inflammasome activation** **in THP1 cells** Western blot analysis of ASC oligomerization in cell lysates of PMA-primed THP1 treated with various doses of carnosol and then stimulated with nigericin. GAPDH served as a loading control in cell lysates.

**Figure S3. Carnosol blocks the production of IL-1β and TNF-α in septic shock mice and experimental NASH model a, b** ELISA of IL-1β and TNF-α in the peritoneal cavity fluid of C57BL/6 female mice treated with vehicle or various doses of carnosol for 1 h and then intraperitoneally injected with LPS (20 mg/kg) for 4 h. **c, d** ELISAs of IL-1β and TNF-α in liver proteins of the NASH mouse model. Data are represented as mean ± SD. The significant differences were analyzed using Mann-Whitney U test: *P < 0.05, **P < 0.01, ***P < 0.001 vs. the control, NS: not significant.
