# Supplementary figures and images for: Carnosol inhibits inflammasome activation by directly targeting HSP90 to treat inflammasome-mediated diseases
Source: Cell Death Dis. 2020 Apr 20;11(4):252. doi: 10.1038/s41419-020-2460-x (PMC7170921; doi:10.1038/s41419-020-2460-x)

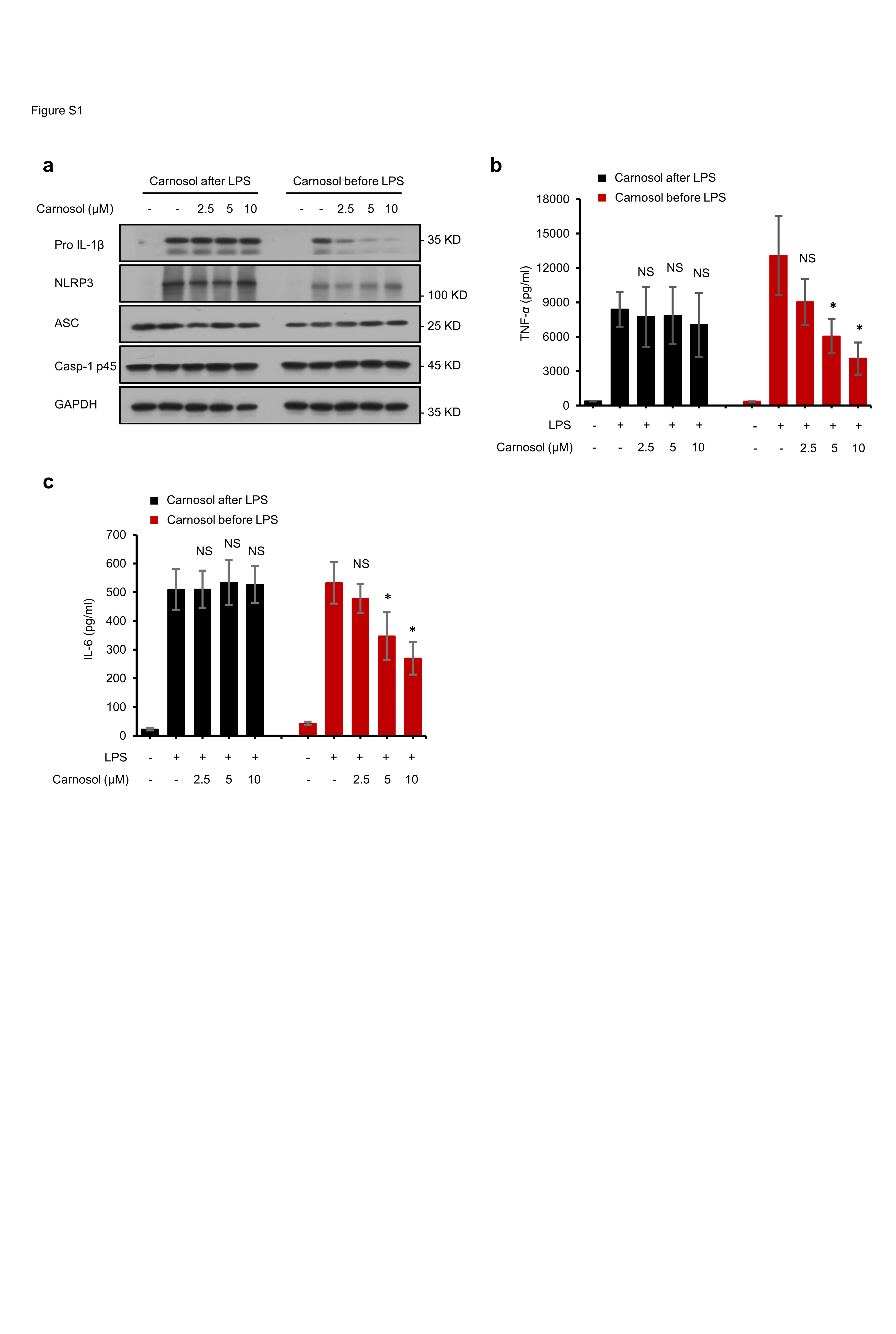

Supplement: Supplementary file 2 — Supplementary Figure 1 [file 41419_2020_2460_MOESM2_ESM.tif]

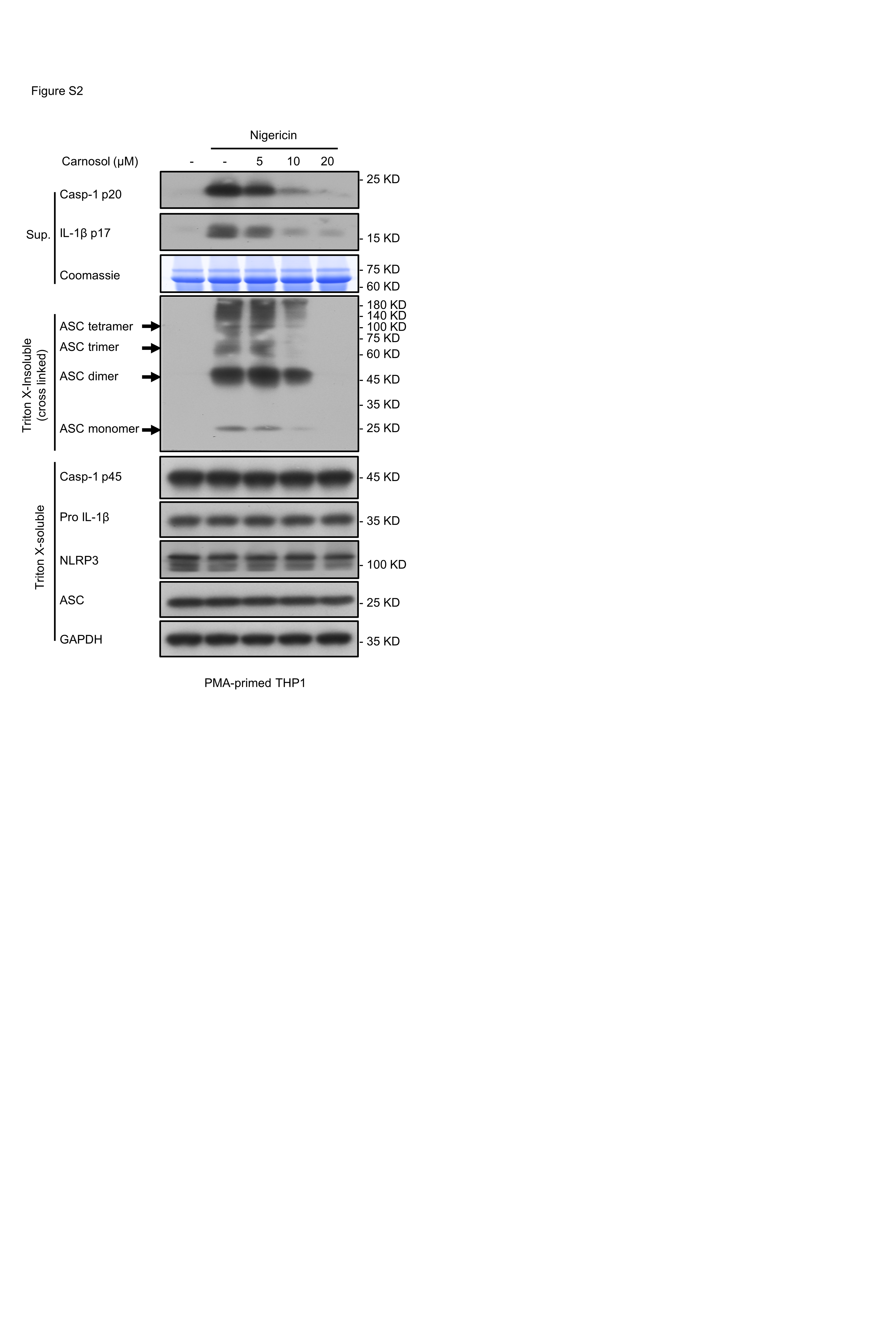

Supplement: Supplementary file 3 — Supplementary Figure 2 [file 41419_2020_2460_MOESM3_ESM.tif]

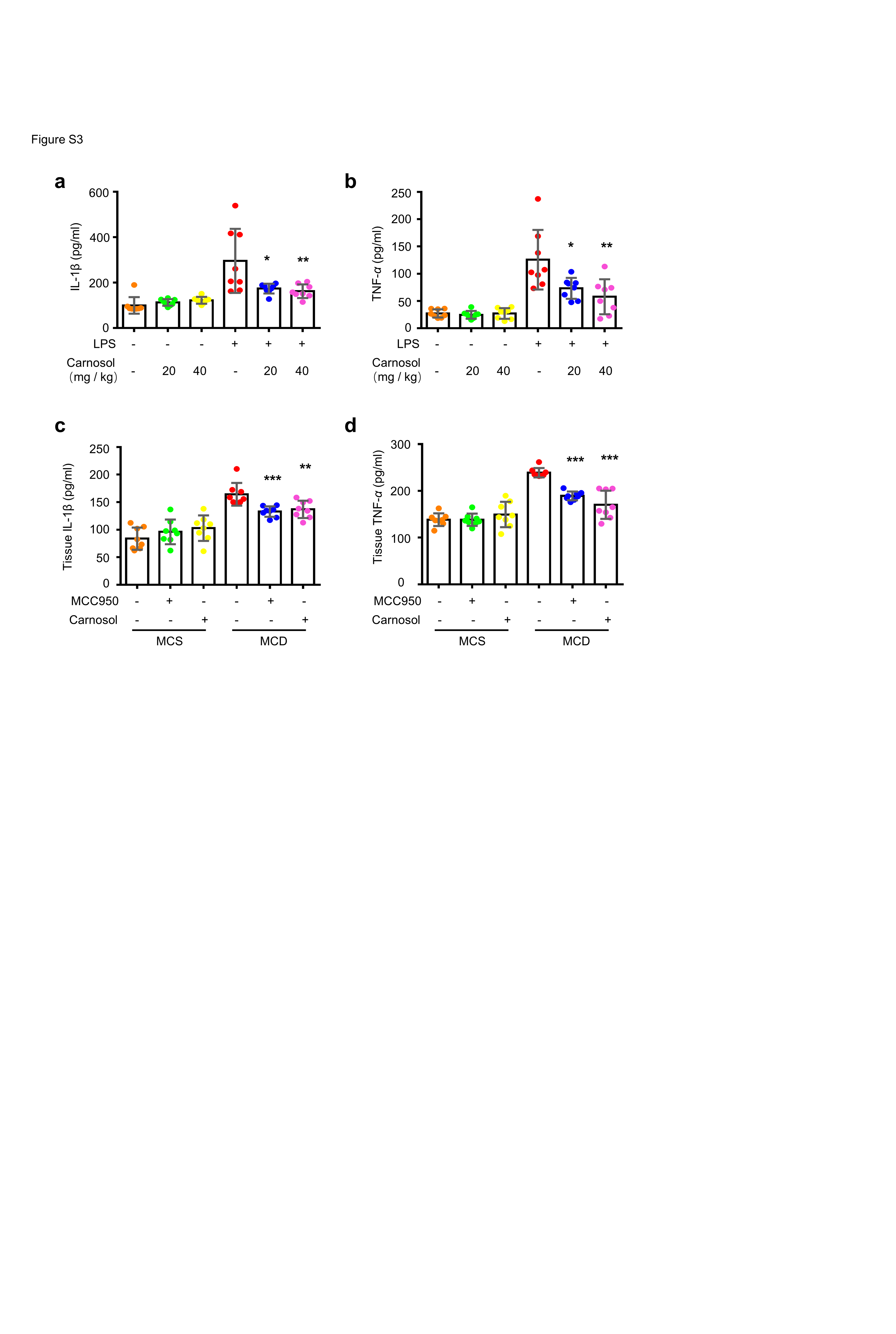

Supplement: Supplementary file 4 — Supplementary Figure 3 [file 41419_2020_2460_MOESM4_ESM.tif]
